# Amylene

**Published:** 1857-07

**Authors:** 


*Amylene.*—We copy the following from the London Medical Times and Gazette.—Eds.

Dr. Kadlburger, (*Wien Wochenschreft, No.* 19,) relates the results of his experience of the employment of amylene in seventy-two cases of tooth extraction, he applying it by means of a sponge to both mouth and nostrils.

He states that the amylene he employs emits a smell of over-ripe pears, and is nowise irritating to the respiratory organs. From one to one and a half minutes inhalation suffices for the purpose. The falling of the uplifted arm, as if wearied, is the signal for the discontinuance of the inhalation. No abnormal muscular movements or enclosure of the jaw were produced. The patient readily opened the mouth upon being requested to do so; and during the extraction most persons retained the consciousness of the application of the instruments. The pain, when felt at all, was very slight, and never at all approaching to the torture attendant upon ordinary tooth drawing.

